# Mycosis Fungoides, Sézary Syndrome, and Cutaneous B‐Cell Lymphomas: 2025 Update on Diagnosis, Risk‐Stratification, and Management

**DOI:** 10.1002/ajh.27735

**Published:** 2025-06-10

**Authors:** Alexandra C. Hristov, Trilokraj Tejasvi, Ryan A. Wilcox

**Affiliations:** ^1^ Department of Pathology University of Michigan Ann Arbor Michigan USA; ^2^ Department of Dermatology University of Michigan Ann Arbor Michigan USA; ^3^ Department of Internal Medicine University of Michigan Ann Arbor Michigan USA

**Keywords:** cutaneous B‐cell lymphoma, cutaneous T‐cell lymphoma, mycosis fungoides, non‐Hodgkin lymphoma

## Abstract

**Disease Overview:**

Primary cutaneous lymphomas are a rare and heterogeneous group of extranodal lymphomas that require the integration of clinical and histopathologic data for classification and treatment.

**Diagnosis:**

Diagnosis and disease classification is based on histopathologic review and immunohistochemical staining of an appropriate skin biopsy. Pathologic review and appropriate clinical and staging evaluations are necessary to distinguish primary cutaneous and systemic lymphomas.

**Risk‐Stratification:**

Disease histopathology remains the most important prognostic determinant in primary cutaneous B‐cell lymphomas, whereas staging remains an important prognostic determinant in cutaneous T‐cell lymphomas.

**Risk‐Adapted Therapy:**

PCFCL and PCMZL patients with solitary or relatively few skin lesions may be effectively managed with local radiation therapy. While single‐agent rituximab may be employed for patients with more widespread skin involvement, multiagent chemotherapy is rarely appropriate. In contrast, the management of patients with PCDLBCL, LT is comparable to the management of patients with systemic DLBCL. Clinical staging forms the basis for a “risk‐adapted,” multi‐disciplinary approach to CTCL treatment. For patients with disease limited to the skin, disease‐specific and overall survival is measured in years, if not decades, and skin‐directed therapies are preferred. In contrast, patients with advanced‐stage disease, including cutaneous tumors or significant nodal, visceral, or blood involvement, are approached with systemic therapies in an escalating fashion. In selected patients, allogeneic stem‐cell transplantation may be considered, as this is curative in some patients.

## 1 Disease Overview

Primary cutaneous lymphomas are a heterogeneous group of extranodal non‐Hodgkin lymphomas that are classified (Table 1) in the updated World Health Organization (WHO) and the International Consensus Classification (ICC) [1, 2, 3]. Most (≈70%) primary cutaneous lymphomas are T‐cell derived, including mycosis fungoides (MF) and Sezary syndrome (SS). In contrast, B‐cell derived cutaneous lymphomas are classified into three major entities: primary cutaneous follicle‐center lymphoma (PCFCL), primary cutaneous diffuse large B‐cell lymphoma, leg type (PCDLBCL, LT), and primary cutaneous marginal zone lymphoma/lymphoproliferative disorder (PCMZL/LPD) [1, 2, 3, 4]. EBV‐positive mucocutaneous ulcer (EBVMCU) is a recently described, EBV+ lymphoproliferative disorder that affects the skin and mucosa. The incidence rates for these primary cutaneous T‐cell lymphomas (CTCL) and primary cutaneous B‐cell lymphomas (CBCL) have been increasing, but are currently < 10 per million persons [5, 6, 7], with the highest incidence rates being observed in males and adults over the age of 50.

**TABLE 1:** WHO and ICC classifications (2022).^a^

WHO	ICC
Cutaneous T‐cell lymphomas and lymphoproliferative disorders	
Mycosis Fungoides Subtypes: Folliculotropic MF (FMF) Pagetoid reticulosis Granulomatous slack skin (GSS)	Mycosis Fungoides Variants: FMF Pagetoid reticulosis
Sézary syndrome	Sézary syndrome
CD30 Lymphoproliferative disorders: Primary cutaneous anaplastic large cell lymphoma (pcALCL) Lymphomatoid papulosis (LyP)	CD30 Lymphoproliferative disorders: pcALCL LyP
Subcutaneous panniculitis‐like T‐cell lymphoma (SPTCL)	SPTCL
Severe mosquito bite allergy Hydroa vacciniforme lymphoproliferatie disorder (LPD)	Severe mosquito bite allergy Hydroa vacciniforme LPD
Primary cutaneous (PC) gamma/delta T‐cell lymphoma	PC gamma/delta T‐cell lymphoma
PC aggressive epidermotropic cytotoxic T‐cell lymphoma	PC aggressive epidermotropic cytotoxic T‐cell lymphoma
PC CD4^+^ small/medium T‐cell LPD	PC CD4^+^ small/medium T‐cell LPD
PC acral CD8+ LPD	PC acral CD8+ LPD
PC PTCL, NOS	
Cutaneous B‐cell lymphomas and lymphoproliferative disorders	
PC marginal zone lymphoma	PC marginal zone LPD
PC follicle center lymphoma (PCFCL)	PCFCL
PC diffuse large B‐cell lymphoma, leg type (PCDLBCL, LT)	PCDLBCL, LT
Intravascular large B‐cell lymphoma	Intravascular large B‐cell lymphoma
EBV+ mucocutaneous ulcer	EBV+ mucocutaneous ulcer

^a^ Adapted from [4].

Genetic evidence implicates UV radiation as a risk factor for CTCL [8, 9, 10], while epidemiological studies suggest that exposure to environmental toxins may confer an increased risk [11]. Selected medications and possibly COVID‐19 vaccination have also been associated with CTCL [12, 13, 14]. Antigen‐ or cytokine‐driven T‐cell lymphoproliferation or dyscrasia may explain these extraordinary and potentially iatrogenic CTCL cases.

Rare reports of familial MF and the detection of specific HLA class II alleles in association with both sporadic and familial MF suggest that host genetic factors may contribute to MF development [15, 16, 17]. Patients with CTCL have a higher incidence of secondary malignancies, including melanoma, meriting appropriate screening and surveillance [18, 19, 20]. Patients with CBCL also have a higher incidence of subsequent cancers, including thyroid, renal, and lung cancer in women, and melanoma, bladder, and prostate cancers in men [21].

The genetic and molecular pathogenesis of both CBCL and CTCL has been informed by next‐generation sequencing efforts, many of which have been recently reviewed elsewhere [22, 23]. Alterations involving lymphocyte‐specific (including antigen‐ and cytokine‐receptor dependent) signaling pathways, epigenetic and chromatin remodeling proteins, and cell cycle regulators are recurrently involved and have therapeutic implications [22, 24]. Disruption of the skin barrier [25] and the skin microbiome [26, 27, 28] are clinically and therapeutically relevant considerations, at least in CTCL, while localized (within the lymphoma microenvironment) and systemic (the “macroenvironment”) immune dysregulation has a pathogenic role in both CBCL and CTCL [22, 29, 30, 31, 32, 33].

## 2 Diagnosis

### 2.1 CBCL

Diagnosis and classification of a CBCL requires an incisional, excisional, or 4–6 mm punch biopsy which includes reticular dermis and subcutaneous fat for morphologic and immunohistochemical analysis, and an appropriate staging evaluation to exclude systemic disease [34]. The use of appropriate immunohistochemical stains (e.g., CD5, cyclin D1) may also aid in distinguishing CBCL from secondary skin involvement by a systemic lymphoma.

#### 2.1.1 PCFCL

PCFCL commonly presents as a solitary purple to pink colored papule or nodule (often biopsied to rule out a non‐melanoma skin cancer) or as multiple papules, plaques, or tumors with a rim of peripheral erythema occurring on the forehead, neck, and upper back in middle‐aged adults. While grouped lesions may be observed, multifocal disease is less common. Other, less common presentations include rhinophyma and scarring alopecia with tumid pink plaques. Dermoscopy may help differentiate these cutaneous lymphomas from more common non‐melanoma skin cancers [35]. Histopathologically, PCFCL is characterized by a follicular, diffuse, or mixed growth pattern comprised of large centrocytes and variable centroblasts derived from germinal‐center B‐cells [36, 37, 38]. In contrast to systemic follicular lymphomas, the majority of PCFCL do not harbor the t(14;18) translocation involving the bcl‐2 locus, and do not strongly express bcl‐2 by immunohistochemistry, although expression may be observed in a minority of cases [39, 40, 41, 42, 43]. Strong expression of bcl‐2 and CD10 may suggest secondary cutaneous involvement by follicular lymphoma, and bcl‐2 expression in > 75% of cells may be associated with an increased risk of skin recurrence in PCFCL [44]. PCFCLs express bcl‐6, variably express CD10, and are MUM‐1/IRF‐4, FOXP1, and IgM negative, consistent with their origin from germinal‐center B cells [42, 45]. Zhou et al. performed whole‐exome sequencing in both PCFCL (*n* = 30) and in systemic FL with secondary cutaneous involvement (*n* = 10) [43]. Consistent with prior observations, most (i.e., 73%) PCFCL did not express bcl‐2, and when compared with secondary cutaneous FL were more proliferative, as determined by Ki67 expression. Notably, recurrent mutations in the epigenetic modifiers CREBBP, KMT2D, EZH2, or EP300 were recurrently observed in secondary cutaneous FL, but were rarely observed in PCFCL. Consequently, mutations in at least two of these genes were observed in 63% of secondary cutaneous FL cases, but were rarely observed in PCFCL [43]. In contrast, TNFRSF14 is frequently mutated in PCFCL [46]. A recent study using low‐coverage whole‐genome sequencing found that systemic FL was more likely to have amplifications of chromosome 18, particularly at the BCL2 locus. In addition, PCFCL with distant spread was associated with genomic instability, amplifications of *REL* and *XPO1*, and deletions of *CDKN2A* and *CDKN2B* [47]. Therefore, the genetic landscape may help distinguish PCFCL from secondary cutaneous FL. A novel variant involving the lower female genital tract (cervix and/or vagina) was recently described [48], sharing immunophenotypic (e.g., > 80% Bcl‐2 negative), genetic (e.g., no CREBBP, KMT2D mutations were identified), and clinical characteristics (localized disease with 100% 5‐year overall survival) with PCFCL. Notably, some cases of PCFCL with a diffuse growth pattern and large centrocytes can be difficult to distinguish from DLBCL. These cases may have a high proliferation index [49, 50]. However, finding areas of follicular growth, cleaved cells compatible with centrocytes, follicular dendritic networks using immunohistochemistry (e.g., CD21, CD23, CD35, SSTR2A, D‐240), numerous T‐cells, and lack of expression of BCL2, MUM1, FOXP1, IgM, and C‐MYC favor PCFCL [42, 45, 51].

#### 2.1.2 PCDLBCL, LT

PCDLBCL, leg type commonly affects elderly women and presents with rapidly progressive red brown to blue tumors involving either one or both lower legs [42, 52]. Tumors may be ulcerated, and larger tumors may be surrounded by smaller satellite lesions. Less common presentations include verrucous and multicolored nodules. Population‐based studies suggest that involvement restricted to the legs is less common than the designation “leg type” would suggest, as involvement of other cutaneous sites is fairly common [42, 53]. These lymphomas are characterized by diffuse sheets of centroblasts and immunoblasts that spare the epidermis but frequently extend deep into the dermis and subcutaneous tissue. In contrast to PCFCL, lymphoma cells highly express bcl‐2, likely due to gene amplification [54, 55], as t(14;18) is not observed in PCDLBCL, LT. Bcl‐2 overexpression, or dual expression of both bcl‐2 and c‐myc, is associated with inferior overall survival compared [55, 56, 57]. C‐myc and bcl‐2 translocations (“double hits”) are observed in < 20% of PCDLBCL, LT [55, 56]. Most cases are MUM‐1/IRF‐4, FOXP1, IgM and bcl‐6 positive, CD10 negative, and have a gene expression profile resembling activated B cells [38, 42]. EBV in situ hybridization (EBER) is negative. Perhaps not surprisingly, the genetic landscape observed in PCDLBCL, LT is similar to that observed in activated B‐cell‐type diffuse large B‐cell lymphoma (ABC‐DLBCL), with NF‐κB‐activating mutations being observed in *CD79B*, *CARD11*, and *MYD88* [58, 59, 60, 61, 62]. Of these, somatic MYD88 L265P mutations appear most common with a prevalence of ≈75%, and are rarely, if ever, observed in PCFCL [45, 50, 55, 59, 60, 61]. MYC rearrangements, observed in ≈30%, have been associated with inferior outcomes [63, 64]. Despite the use of somatically hypermutated immunoglobulin heavy‐chain variable (IGHV) regions, Staphylococcal superantigen binding sites within the IGHV are preserved, thus implicating superantigen‐dependent B‐cell receptor signaling in disease pathogenesis [65].

#### 2.1.3 PCMZL/LPD

Patients with PCMZL/LPD frequently present with solitary asymptomatic pink papules on the trunk, arms, and head and neck area, or as red‐brown multifocal papules, plaques, or nodules involving the trunk and arms. While an association with *Borrelia burgdorferi* has been observed in Europe, a similar association has not been observed in cases from the United States [66, 67, 68]. PCMZL/LPD are composed of a variably mixed infiltrate of small, “centrocyte‐like” marginal zone B cells, monocytoid B‐cells, lymphoplasmacytic cells, plasma cells, cells resembling centroblasts and immunoblasts, reactive T cells, eosinophils, and histiocytes. Marginal zone B cells characteristically express bcl‐2 and may express CD43 but lack bcl‐6, CD10, CD5, and CD23 expression. Recently, two groups of PCMZL/LPD have been identified [69, 70, 71]. The majority of PCMZL/LPD express class‐switched immunoglobulin and have an indolent course. A recent classification system recommends that this type of PCMZL should be considered a lymphoproliferative disorder (LPD) to reflect its clinical and histopathologic overlap with atypical reactive B‐cell infiltrates (pseudo B‐cell lymphomas) and extremely favorable prognosis [4]. This group is characterized by collections of B‐cells with peripheral plasma cells, numerous intermixed T‐cells and reactive follicles, and may represent a response to an antigenic stimulus [72, 73]. A second group expresses IgM and shows a behavior similar to extracutaneous extranodal marginal zone lymphomas, with frequent recurrences and extracutaneous spread. This group often shows a diffuse infiltrate with intermixed, rather than peripheral, plasma cells and frequent follicular colonization and is more likely to involve the subcutis [72], and more likely to undergo transformation to diffuse large B‐cell lymphoma [74]. PCMZL/LPD often harbor *FAS, SLAMF1*, *SPEN*, and *NCOR* mutations and *IGH* translocations involving *FOXP1* and *BCL10* [75, 76]. Some PCMZL/LPD show overlapping clinical and histopathologic features with primary cutaneous CD4‐positive small or medium lymphoproliferative disorder [51, 77, 78]. These cases have a predominance of small to medium lymphoid cells and may show increased PD‐1 and CD4‐positive T‐cells as well as clonal rearrangements of IGH and TRG. Recurrences were more likely in PCMZL, and mutations in *TNFAIP3* and *FAS* were not identified in primary cutaneous CD4‐positive small/medium lymphoproliferative disorder [77].

#### 2.1.4 EBVMCU

EBVMCU typically presents as a solitary, painful, and well‐circumscribed ulcer on the skin or mucosa, the latter including the oropharynx, genital mucosa, or the gastrointestinal tract [79, 80, 81, 82, 83, 84, 85, 86]. Patients are generally immunosuppressed, either due to age‐related immunosenescence, iatrogenic immunosuppression (particularly with methotrexate), solid organ transplantation, HIV, or primary immunodeficiency. They do not have other symptoms and are without bone marrow involvement, lymphadenopathy, or hepatosplenomegaly. In addition, EBV is not increased in the peripheral blood [83]. Lesions may be associated with preceding trauma, and trauma‐associated inflammation may contribute to pathogenesis. Tissue damage and necrosis with fibrinous changes may lead to an immune‐sequestered area and localized EBV proliferation [86]. Histopathologically, EBVMCU demonstrates a shallow ulcer with an underlying mixed infiltrate that includes numerous plasma cells, histiocytes, and eosinophils and a base composed of numerous CD8+ T‐cells [79, 80, 81, 82, 83, 84]. Scattered throughout, there are variably sized B‐cell immunoblasts, some with a Hodgkin‐Reed‐Sternberg‐like (HRS‐like) appearance. Apoptotic plasmacytoid cells may also be present [79, 84]. Immunohistochemical studies demonstrate that the immunoblasts are non‐germinal center B‐cells that express CD20, PAX5, OCT2, and MUM‐1 and are negative for CD10. They are CD30 positive and may express CD15. Plasma cells may be light chain restricted [80]. EBV in situ hybridization (EBER) marks the immunoblasts, including the HRS‐like cells, as well as smaller lymphoid cells [85]. Angioinvasion and necrosis are often present. B‐cell receptor gene rearrangement studies show a clonal B‐cell population in less than half of cases; T‐cell receptor gene rearrangement studies may be oligoclonal or clonal [79, 80, 81, 83]. The histopathologic differential diagnosis for EBVMCU includes both EBV+ DLBCL and Hodgkin lymphoma, as well as polymorphic lymphoproliferative disorders; clinical features are essential for arriving at the correct diagnosis, especially in small biopsies [80, 87]. A recent next‐generation sequencing study showed that EBVMCU, like EBV+ DLBCL, showed a wide array of gene variants. TET2 mutations associated with clonal hematopoiesis are commonly observed [85].

### 2.2 CTCL

#### 2.2.1 Mycosis Fungoides

The definitive diagnosis of MF, particularly patch/plaque stage disease, is challenging, as many of its clinical and pathologic features are non‐specific and overlap with reactive processes. Many patients will have had symptoms attributed to eczema, psoriasis, or parapsoriasis for years prior to obtaining a definitive diagnosis [88]. The median time from symptom onset to diagnosis in retrospective series is 3–4 years but may exceed four decades [88, 89, 90, 91, 92]. During this time, patients may be misdiagnosed as, and in some cases treated for, other entities, particularly atopic dermatitis and psoriasis [93]. Given the importance of clinicopathological correlation in the diagnosis of MF and the variable association of specific histopathologic findings with the diagnosis, biopsy reports are not infrequently “suggestive of” the diagnosis. This occasional uncertainty implied in biopsy reports and apparent lack of a more definitive histopathologic diagnosis may be a source of frustration for clinicians unfamiliar with the challenges associated with rendering a pathologic diagnosis of MF. Furthermore, treatment with skin‐directed therapies at the time of biopsy, including topical corticosteroids, may diminish or eliminate neoplastic T‐cells and other histopathologic findings, further compounding the diagnostic challenge, as these therapies diminish or eliminate neoplastic T cells and critical histopathologic findings for 2–4 weeks [94, 95]. Drug reactions, chronic spongiotic dermatitis, connective tissue diseases, lichen sclerosus et atrophicus, and pigmented purpuric dermatoses are just a few of the conditions that may mimic MF [96, 97]. While a definitive diagnosis of MF may be made based on clinical and histopathologic features alone, determination of T‐cell clonality and assessment for the aberrant loss of T‐cell antigen (e.g., CD2, CD5, CD7) expression by immunohistochemistry are useful. PCR‐based methods are able to detect clonal rearrangements of the T‐cell receptor (TCR) in formalin‐fixed, paraffin‐embedded biopsy specimens [98, 99]. PCR‐based methods, while sensitive, should be interpreted with caution, as clonal TCR gene rearrangements may be detected in normal elderly individuals and in patients with benign dermatoses or other disease states [100, 101, 102, 103, 104]. However, detection of identical clones from two different sites is quite specific for MF [105]. Even this feature is not without complications as rare reactive processes display what appears to be an identical T‐cell clone by PCR‐based gene rearrangement studies in multiple biopsies over time. Moreover, some MF cases may not have a detectable T‐cell clone [106]. Recent studies have suggested that next generation sequencing (NGS) may be more sensitive and/or specific for assessing T‐cell clonality in MF/SS, but NGS is not yet widely available [107, 108, 109, 110, 111, 112]. Moreover, NGS may have similar pitfalls to PCR‐based studies, as it may identify clonal T‐cells in reactive infiltrates and may not identify clonal T‐cell in CTCL [106, 113]. The extent to which MF/SS may be preceded by a pre‐malignant state, analogous to monoclonal B‐cell lymphocytosis (MBL) or monoclonal gammopathy of undetermined significance (MGUS), is debatable and poorly defined [114]. The malignant lymphocytes in MF/SS are usually CD3^+^CD4^+^ and CD8^−^ but frequently lose the expression of other pan‐T‐cell antigens. Therefore, demonstration of a significant population of CD4^+^ cells lacking CD2, CD5, and/or CD7 expression is highly specific (specificity > 90%) for MF in most reported series [115, 116]. However, reactive dermatoses may also show a predominance of CD4‐positive T‐cells and loss to diminished expression of CD7, the T‐cell antigen most frequently lost in MF, and these results must be interpreted with caution [97, 116]. Finding a marked predominance of CD4‐positive T‐cells, especially by epidermotropic T‐cells, helps to support a diagnosis of MF [97, 116]. Similarly, finding extensive loss of CD7, preferential loss of pan T‐cell antigens by epidermal T‐cells, or loss of multiple pan T‐cell markers favors a diagnosis of MF in challenging cases [97, 116]. Clinically, patch/plaque stage MF is frequently characterized by persistent and progressive lesions that develop in a “bathing suit” distribution and vary in size, shape, and color. These lesions are frequently large (> 5 cm), pruritic, and multifocal in “classical” MF. In skin of color (SoC), lesions are polychromic, including hyper‐ and hypopigmented patches/plaques. Espinosa et al. identified hyperpigmentation, lichenification, and a silver hue as significantly more common in SoC [117]. However, a broad range of MF variants have been described with differences in tropism (e.g., follicular MF, adnexotropism), distribution (e.g., palmoplantar MF), appearance (e.g., slack skin, poikiloderma), pigmentation (e.g., hypo‐ and hyperpigmented variants) and focality (e.g., unilesional MF) [36, 118]. Histopathologically, patch/plaque MF is characterized by enlarged, epidermotropic lymphocytes with irregular nuclei that often show a band‐like distribution in the dermis, where they are associated with dense strips of collagen (“wiry” fibrosis). Aggregates of neoplastic T‐cells in the epidermis, termed Pautrier microabscesses, are seen in a minority of cases but are a helpful clue to the diagnosis. Folliculotropism and/or syringotropism may be seen in a minority of cases. Recent studies have demonstrated that the inclusion of clinical information, including photographs, improved the diagnostic accuracy of pathologists, thus highlighting the importance of clinical information for accurate histopathologic diagnosis [119, 120].

#### 2.2.2 Sézary Syndrome

Traditionally, SS is defined as a leukemic form of CTCL associated with erythroderma, intractable pruritus, ectropion, and palmoplantar keratoderma. A series of studies in the early to mid‐20th century, beginning with Sezary's initial landmark observation in 1938, identified a population of large lymphocytes in the peripheral blood with grooved, lobulated (i.e., “cerebriform”) nuclei in patients with MF or SS [121, 122, 123, 124, 125, 126]. As in other chronic lymphoproliferative disorders, the Sezary cell count is preferably expressed in absolute terms, with ≥ 1000 cells/μL classified as B2 disease in the current ISCL/USCLC/EORTC TNMB staging classification [127], although this exclusive reliance on defining disease burden in absolute terms may misclassify (understage) a minority of patients [128]. The morphologic detection of Sezary cells in the peripheral blood is not specific for CTCL, as Sezary cells may be found in peripheral blood from normal donors and in benign conditions [129, 130, 131]. The histopathologic findings in the skin often resemble those observed in MF, with less prominent epidermotropism, though findings in skin biopsies may be paradoxically subtle and non‐specific. As in MF, immunohistochemical studies showing a CD4 predominance and loss of pan T‐cell markers may be helpful. Lymph node involvement is characterized by complete effacement of the nodal architecture by infiltrating Sezary cells [132].

In SS, clonal T cells are generally CD3^+^CD4^+^ and CD8^−^ by multi‐color flow cytometry [133, 134, 135, 136]. As in MF, the aberrant loss of pan‐T‐cell antigens, including CD2, CD3, CD4, CD5, CD7, and/or CD26, is frequently observed [135, 137, 138, 139, 140]. Of these, the aberrant loss of CD7 and/or CD26 expression is most common, being observed in most cases [136, 137, 141, 142, 143, 144, 145]. Loss of both CD7 and CD26 expression in > 40% of peripheral blood CD4^+^ T cells is 100% specific for SS [146]. Molecular studies, including detection of a clonal TCR gene rearrangement by PCR and the presence of a clonal cytogenetic abnormality, provide evidence of T‐cell clonality. An alternative approach to demonstrate T‐cell clonality incorporates multi‐color flow cytometry using a panel of antibodies specific for various TCR beta‐chain variable region family members (TCR‐Vβ) [147, 148, 149]. This approach is successful in identifying a clonal population of T cells if this population is significantly higher than the background frequency of polyclonal T cells harboring the same Vβ chain [147, 148, 150]. The beta‐chain constant region includes two gene segments (C1 and C2). In a manner analogous to kappa or lambda light chain restriction in B‐cell lymphoproliferative disorders, over (or under) representation of beta‐chain constant chain‐1 region (TRBC1) is a sensitive and specific biomarker for αβ T‐cell clonality [151, 152, 153]. Detection, by FISH, of driver genes that are recurrently amplified or deleted in CTCL is informative but not widely available [154, 155].

In addition to the ISCL/USCLC/EORTC criteria, the most recent WHO classification requires erythroderma, generalized lymphadenopathy, and clonally related T‐cells (Sézary cells) in the skin, peripheral blood, and lymph nodes. On rare occasions, SS may be preceded by a prior history of patches/plaques, compatible with classic MF. The ISCL recommends that such cases be designated as SS preceded by MF or secondary erythrodermic CTCL Conversely, patients with MF, but without erythroderma, may meet hematologic criteria for SS. In these cases, the designation “MF with leukemic involvement” is recommended.

#### 2.2.3 Non‐MF/SS Subtypes of CTCL

An important goal during a patient's initial diagnostic evaluation is to distinguish non‐MF/SS CTCL subtypes from MF/SS, as the natural history, prognosis, and treatment approach for each of the non‐MF/SS lymphomas is highly variable. A detailed description of these CTCL subtypes is beyond the scope of this update, but the salient features of each have been previously summarized [2, 156, 157].

## 3 Risk‐Stratification

### 3.1 CBCL

Staging recommendations include a history, physical examination, and imaging (either CT, PET, or increasingly PET/CT) [158, 159, 160]. A bone marrow biopsy and aspirate should be performed in cases of PCDLBCL, LT, and in patients with other CBCL with otherwise unexplained cytopenias [158]. While the TNM staging classification describes the extent of disease, staging in CBCL is of limited prognostic value, as the disease histopathology is the major determinant in risk stratification. This is highlighted by a population‐based study, which identified histopathology and the site of skin involvement as important prognostic factors [161]. In contrast, the International Extranodal Lymphoma Study Group identified three independent prognostic factors (i.e., elevated LDH, > 2 skin lesions, and nodular lesions) among patients with PCFCL and PCMZL. These factors were combined to form the cutaneous lymphoma international prognostic index (CLIPI). The absence of any adverse prognostic factor was associated with a 5‐year progression‐free survival of 91%. In contrast, the presence of two or three adverse prognostic factors was associated with a 5‐year progression‐free survival of 48%. As the vast majority of relapses were confined to the skin, the CLIPI was unable to risk stratify patients by overall survival. The presence of multiple skin lesions was associated with inferior disease‐free survival in a European series [162], but was not associated with disease‐free survival in a large North American series [163]. The most important factor for risk stratification among the cutaneous B‐cell lymphomas remains the histopathologic classification. Indolent CBCL (PCFCL and PCMZL) are associated with 5‐year disease‐specific survival ≥ 95% [36, 163], though PCMZL with IgM expression has been associated with a less favorable course. Neither the presence of a matching B‐cell clone in the skin and the blood nor a paraproteinemia is associated with cutaneous relapse or prognosis in PCMZL/LPD [164]. Differences in growth pattern, the density of centroblasts, and cytogenetic findings do not appear to provide meaningful prognostic information in PCFCL. While rare, a subset of systemic FL may initially present with cutaneous involvement, but with extracutaneous disease that is below the threshold of radiographic detection. The presence of mutations in *CREBBP*, *KMT2D*, *EZH2*, *EP300* (as observed in systemic FL), rearrangements involving the bcl‐2 gene, and a low proliferation rate (Ki67 < 30%) may identify these patients [43]. In contrast, PCDLBCL, LT is associated with a 5‐year disease‐specific survival of approximately 50%, and dual bcl‐2 and c‐myc expression [36, 45, 55, 59, 165] and loss of *CDKN2A* are associated with inferior survival [166]. The presence of a somatic MYD88^L265P^ mutation is also associated with inferior disease‐specific and overall survival [59]. In contrast to patients presenting with only a single tumor, involvement of multiple sites, on one or both legs, is associated with a significantly inferior disease‐specific survival [42, 167].

While EBVMCU may spread or recur locally, distant dissemination is exceedingly rare, and if observed, ought to prompt consideration of alternative EBV‐associated lymphoproliferative neoplasms. Thankfully, EBVMCU are generally localized and associated with a favorable prognosis, as most cases regress following a reduction in immunosuppression [168, 169].

### 3.2 CTCL

In contrast to many other lymphoproliferative disorders in which cytogenetic and laboratory findings play a prominent role in risk stratification, TNMB (tumor, node, metastasis, blood) staging remains an important prognostic factor in MF/SS and forms the basis for a “risk‐adapted” approach to treatment. Patients with only patches and plaques have stage I disease, but may be further divided into stage IA (< 10% body surface area involved or T1) or stage IB (> 10% body surface area involved or T2) based on the extent of skin involvement, and by the presence of patch‐ (T1a/T2a) or plaque‐stage (T1b/T2b) disease [158]. For practical purposes, the area of a patient's hand (including both palm and digits) represents approximately 1% of body surface area. Current staging and diagnostic recommendations do not require a biopsy of clinically normal lymph nodes; however, an excisional or core‐needle biopsy of any abnormal lymph nodes (≥ 1.5 cm in diameter or firm/fixed) is recommended, with preference being given either to the largest lymph node draining an area of skin involvement or to the node with the greatest standardized uptake value (SUV) on FDG‐PET imaging [170, 171, 172]. While radiologic examination of lymph nodes may be optional for patients with T1 or T2 disease and no evidence of lymphadenopathy on physical examination [173], a recent international study found that physical examination may miss radiographically‐enlarged lymph nodes leading to significant changes in staging in a minority of patients, particularly those with plaques [174]. Patients with patch/plaque stage disease (T1/T2) and architectural preservation of any clinically abnormal lymph nodes are classified as stage IIA. Collectively, patients with stage I‐IIA disease have “limited (or early)‐stage” disease, as the overall survival in these patients is measured in decades, with survival in patients with stage IA disease resembling that of normal age‐matched controls [89, 90, 175]. At diagnosis, the majority of MF patients will have limited‐stage disease [175]. In contrast, patients with tumor stage disease (T3), erythroderma (T4), nodal involvement characterized by partial or complete architectural effacement (N3), visceral metastases (M1), or significant leukemic involvement (B2) have “advanced (or late)‐stage” disease. While the European Society for Medical Oncology (ESMO) and the EORTC have recommended peripheral blood flow cytometry for all MF/SS patients [146, 176], recent consensus recommendations support of the use of peripheral blood flow cytometry in specific patient groups: those with advanced‐stage (≥ IIB) disease, intractable pruritis, generalized patches/plaques, erythroderma, lymphocytosis, an elevated LDH, or a lack of response to skin‐directed therapies [177]. Unfortunately, median survivals from approximately 1–5 years are observed in these patients with more extensive disease [175]. Large‐cell transformation (LCT), defined as > 25% involvement by aberrant T cells that are at least 4 times greater in size than normal lymphocytes, is genomically complex [30], transcriptionally distinct [178], and is associated with poor outcomes [175, 179].

A retrospective study including 1398 MF patients, 71% with patch/plaque stage disease, and 104 SS patients has validated staging classification [175], as the TNMB classification is significantly associated with overall and disease‐specific survival. A more recent meta‐analysis reported a similar trend for 5‐year survival [180]. While the impact of recently approved agents on overall survival is uncertain, the rather durable responses observed in subsets of patients treated with these agents may provide ample reason for optimism. For those with early‐stage disease, male gender, age > 60, plaque‐stage or folliculotropic disease, and nodal stage N1/Nx were adverse prognostic factors and were utilized to generate the cutaneous lymphoma international prognostic index (CLIPi) for patients with early‐stage disease [181]. Ten‐year OS was 90.3% for those with low‐risk (0–1 risk factors) disease and 48.9% for those with high‐risk (3–5 risk factors) disease. Similarly, male gender, age > 60, stage B1/B2 or N2/N3 disease, and visceral involvement were adverse prognostic factors for patients with late‐stage disease. Ten‐year OS was 53.2% for low‐risk patients, and 15.0% for high‐risk patients [181]. In a large, international series (*n* = 1275) of late‐stage MF/SS, stage IV disease, age > 60, LCT, and elevated LDH were identified as independent adverse prognostic factors and were similarly combined in a prognostic index [182]. Patients with low‐risk (0–1 risk factors) disease experienced superior 5‐year OS (68%) compared with the 5‐year OS observed (28%) among those with high‐risk (3–4 risk factors) disease. An alternative staging system has been proposed for those with folliculotropic MF and identifies a subset of patients with limited cutaneous involvement and a more favorable prognosis [183, 184]. Given the importance of the TNMB classification in risk stratification and defining disease burden, its use in defining the initial, maximum, and current burden of disease, which will ultimately play an important role in the selection of either skin‐directed or systemic therapies, is recommended [158, 173]. In the future, it is anticipated that improved understanding of the genetic landscape will further improve risk stratification and lead to a more personalized approach for treatment selection in CTCL [8].

## 4 Treatment

### 4.1 CBCL

As no randomized controlled trials are available, treatment recommendations for CBCL are largely based on small retrospective studies and institutional experience. The EORTC and ISCL have published consensus treatment recommendations that are consistent with NCCN guidelines [185]. In most cases, optimal patient management requires a multidisciplinary approach, including dermatology, medical oncology, and radiation oncology.

#### 4.1.1 PCFCL

For patients with solitary lesions, low‐dose radiation therapy is safe and highly affective, with a complete remission rate approaching 100%. Radiation does not appear inferior to multiagent chemotherapy among patients with multiple lesions that can be included in multiple radiation fields [186]. In a large North American series, the rate of local control for indolent CBCL with radiation alone was 98% [163]. In the same series, a local recurrence requiring radiation therapy was observed in 25% of patients who had undergone surgical excision alone. Reserving radiation until disease recurrence did not appear to compromise disease‐specific or overall survival [163]. Therefore, complete excision alone, deferring radiation until disease recurrence, is also reasonable. Intralesional (e.g., corticosteroids or rituximab [187, 188, 189]) or topical therapies including corticosteroids, nitrogen mustard and bexarotene may also be considered [190, 191]. While radiation therapy is generally recommended for patients with a solitary lesion, radiation therapy or observation (i.e., “watch and wait”) are reasonable options for those patients with multiple lesions. Rarely, patients with PCFCL may show a locally aggressive course and some have suggested the possibility of transformation to DLBCL [192, 193], suggesting that “watch and wait” patients require close clinical follow‐up. Patients with more extensive skin involvement are effectively managed with single‐agent rituximab [185]. Approximately one‐third of patients may relapse following either radiation or single‐agent rituximab, but relapses are usually confined to the skin and are approached in a manner similar to that described for the initial management of PCFCL.

#### 4.1.2 PCMZL/LPD

Patients with PCMZL are approached in a manner analogous to that described in the initial management of PCFCL. Radiation therapy or surgical excision is associated with high response rates for patients with a single or few lesions [185]. However, some studies have found an increase in recurrence in patients who have undergone surgical excision, as compared to radiation therapy [194, 195]. Those with more widespread skin involvement may be observed. Once symptomatic, culprit lesions may be irradiated or surgically excised [196]. As for PCFCL, single‐agent rituximab may be utilized in patients with symptomatic, widespread skin lesions. An initial trial of antibiotics for those with *B. burgdorferi* ‐associated PCMZL has been recommended [197] but is less relevant for North American patients.

#### 4.1.3 PCDLBCL, LT

As previously noted, the natural history of PCDLBCL, LT more closely resembles that of systemic DLBCL. Therefore, R‐CHOP (with or without radiation therapy) is utilized in these patients. While few reports are available in the literature, the use of R‐CHOP in these patients is associated with disease‐free survival rates rivaling those reported for patients with high‐risk systemic DLBCL [42, 52, 163, 185]. Many patients present with disease confined to a single site and are managed like patients with limited‐stage systemic DLBCL with R‐CHOP and involved field radiation therapy. The management of relapsed disease is comparable to that for relapsed systemic ABC‐DLBCL (e.g., lenalidomide [198], ibrutinib [199], or tafasitamab/lenalidomide [200]). In a small phase II study (*n* = 19), the 6‐month overall response rate with single‐agent lenalidomide in relapsed/refractory PCDLBCL, LT was 26% but was significantly higher in patients without the MYD88^L265P^ mutation [201]. The efficacy of checkpoint (PD‐1) blockade was recently suggested, as 3 out of 5 patients with relapsed/refractory disease achieved durable responses upon PD‐1 blockade [202].

#### 4.1.4 EBVMCU

Among EBVMCU associated with immunosuppression, the vast majority spontaneously regress upon discontinuation or reduction of immunosuppression. For those associated with age‐related immunosenescence and those with a relapse and remitting course following reduction of immunosuppression, either focal radiation therapy or single‐agent rituximab is highly effective [168, 169].

### 4.2 CTCL

#### 4.2.1 Treatment of Limited‐Stage MF

**FIGURE 1:**
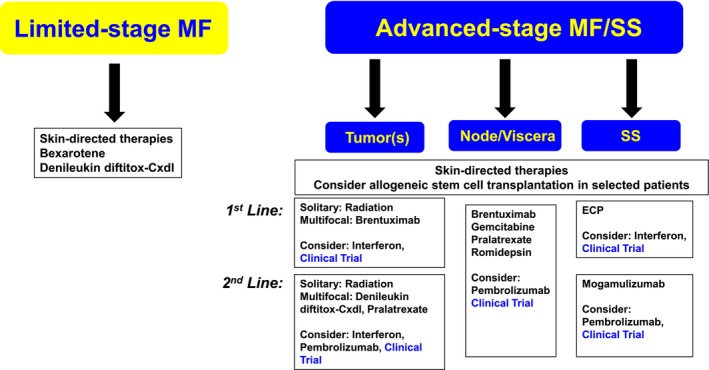
Treatment considerations in MF/SS by stage and disease compartment involvement.

As the majority of CTCL patients present with patch/plaque stage MF and have an excellent prognosis, the initial goal of therapy is to improve symptoms and quality of life while avoiding treatment‐related toxicity. For many patients, this may involve either expectant management (i.e., “watch and wait”) or skin‐directed therapies (Figure 1). Systemic therapies do not increase response rates or survival in these patients [203, 204]. An international prospective study compared skin‐directed therapies (topical steroids, ultraviolet B, psoralen and ultraviolet A, topical nitrogen mustard, topical carmustine and local radiotherapy) to systemic therapy (oral retinoids, oral bexarotene, methotrexate, interferon and extracorporeal photochemotherapy) in early‐stage MF. Patients receiving skin‐directed therapy had a superior overall response rate [204]. The limited efficacy associated with chemotherapy has been highlighted in retrospective studies in which the median time to next treatment following single or multiagent chemotherapy was ≤ 4 months [205, 206]. Therefore, patients with limited‐stage disease who require therapy are best approached with skin‐directed therapies, usually under the direction of a dermatologist and/or radiation oncologist. Excellent reviews and treatment guidelines are available [207, 208].

#### 4.2.2 Topical Therapies

The first‐line treatment for limited stage MF is topical steroids. In an uncontrolled prospective study, topical clobetasol propionate was used in 85% of patients with stage 1A/B disease, had an overall response rate of 94%, and is associated with minimal to no toxicity [209, 210]. An alternative topical medication is mechlorethamine 0.02% gel [211]. In a phase 2 trial, patients with stage IA‐IIA MF were treated with 0.02% gel daily for up to 12 months. A response was observed in 58.5% of patients, with 13.8% achieving a complete response. A sustained response was observed in 85.5% of patients and the most common adverse effects are contact dermatitis and irritant dermatitis [212]. For refractory and persistent cutaneous lesions, bexarotene 1% topical gel may be considered. Prospective trials have demonstrated an ORR between 44% and 63% [213]. Topical toll‐like receptor (TLR) agonists, which lead to local production of interferons and other cytokines, induce cell death and promote host anti‐tumor immunity [214], and have demonstrated efficacy in limited stage MF. For example, 20 patients with stage 1A‐2B disease were treated with 5% Imiquimod, a TLR7 agonist, and an ORR of 80%, including 45% complete responses, were observed. Toxicities are limited, including localized pain, redness, ulceration, and pruritus. Systemic symptoms, including flu‐like symptoms and fatigue, while reported, are rare. Most adverse events are self‐limited and resolve after the first few weeks of treatment [214, 215]. Resiquimod, a potent TLR7/8 agonist, was examined in a phase 1 trial using 0.03 and 0.06% topical resiquimod gel. Among the 12 patients treated, clinical improvement was observed in 75% of treated lesions and 90% of patients had a reduction in malignant T cell clones in the treated lesions, and an abscopal and presumably immune‐mediated effect was observed [216].

#### 4.2.3 Phototherapy

Phototherapy is an important treatment modality that may be used alone or in combination with topical therapies in patients with limited‐stage disease, and includes narrowband UVB (NBUVB, 311 nm) and 8‐methoxypsoralen plus ultraviolet A (PUVA). NBUVB is used in both patch and plaque stage MF. PUVA is the modality of choice in skin of color. Phototherapy is widely available and has demonstrated efficacy in many retrospective and prospective studies [95, 217], and a comprehensive consensus statement on the use of phototherapy was recently published [218].

#### 4.2.4 Radiation

MF/SS are radiosensitive, thus radiation therapy, with curative intent, may be considered in patients with localized, unilesional MF. For those with more widespread disease, palliative local radiation or low‐dose total skin electron beam therapy (TSEBT) are effective ([219], reviewed in [220], [221]).

### 4.3 Treatment of Advanced‐Stage MF/SS

#### 4.3.1 Overview

Patients with advanced‐stage MF/SS require a multidisciplinary approach, as various combinations of skin‐directed therapies, biologic‐response modifiers, and ultimately the sequential use of systemic agents are frequently employed in the management of these patients (Figure 1). As for limited‐stage disease, multiagent chemotherapy is not appropriate [203]. Instead, a “risk‐adapted” and stage‐based approach, consistent with NCCN guidelines, incorporating biologic‐response modifiers (e.g., bexarotene and interferon‐alpha), histone deacetylase inhibitors (e.g., romidepsin), or monoclonal antibodies or antibody‐drug conjugates (e.g., mogamulizumab, brentuximab vedotin) is generally preferred [222]. Therapeutic decisions are individualized and based on a patient's age, performance status, extent of disease burden, the rate of disease progression, and previous therapies [223, 224, 225, 226, 227, 228]. Despite widely disparate prescribing patterns globally, differences in survival have not been observed [229]. The tissue compartment(s) (i.e., skin, blood, lymph node, and/or viscera) involved and the tissue compartment(s) in most urgent need of treatment are also important factors in therapeutic decision making, as many agents are not equally effective across tissue compartments. For example, in patients with tumor‐stage disease, but without blood, nodal, or visceral involvement (stage IIB), the overall response rate observed with brentuximab vedotin was 40%, compared to response rates ≤ 15% in patients with more advanced (> stage IIB) disease [230, 231]. In contrast, the overall response rates observed with mogamulizumab in the blood, skin, and nodes/viscera compartments were 68%, 42%, and ≤ 17%, respectively [232].

#### 4.3.2 Bexarotene

The endogenous retinoids all‐*trans* retinoic acid and 9‐*cis* retinoic acid (i.e., vitamin‐A‐derived compounds) regulate a diverse array of biologic processes, ranging from embryonic development to cell growth, differentiation, and survival, upon binding two families of steroid hormone receptors, the retinoic acid receptors (RAR) and retinoid X receptors (RXR). Upon forming homo‐ or heterodimers, these receptors recruit various nuclear co‐repressor or co‐activator proteins depending on whether or not they are bound by ligand. Multiple RAR retinoids have been used in MF/SS, either topically or systemically (reviewed in [233, 234]), with response rates exceeding 50%. However, in 1999, the oral RXR‐selective “rexinoid” bexarotene was FDA approved for CTCL and was later approved as a topical gel formulation. Laboratory studies demonstrate that bexarotene promotes cell cycle arrest and apoptosis in CTCL cell lines [235, 236]. In a multicenter phase II‐III study, 94 patients with advanced‐stage CTCL who had been previously treated with a median of five prior therapies, the vast majority of whom had disease refractory to at least one prior systemic therapy, received at least 300 mg/m^2^ of oral bexarotene daily [237]. Among patients treated at the 300 mg/m^2^ dose, an overall response rate of 45% was observed, only 2% of which were complete. While an improved overall response rate was noted with the use of higher doses, this difference was not statistically significant, and dose‐limiting toxicity was far more common (50% vs. 89%) in these patients. While a dose–response relationship is likely, the 300 mg/m^2^ dose appears to provide the optimal risk–benefit ratio. The most common toxicities associated with therapy were hypertriglyceridemia (in 82%) and central hypothyroidism (29%). Myelosuppression is infrequent and usually uncomplicated. Pancreatitis secondary to hypertriglyceridemia may be rarely observed but is reversible upon discontinuation of treatment. Therefore, a baseline lipid panel and free T4/TSH should be obtained prior to the initiation of therapy. In one retrospective study, all patients treated with bexarotene developed hyperlipidemia and central hypothyroidism, frequently within weeks of initiating treatment [238]. Consequently, use of lipid‐lowering agents (e.g., fenofibrate) and low‐dose levothyroxine (e.g., 50 micrograms) prior to initiating bexarotene is generally recommended [239, 240, 241]. In clinical practice, bexarotene is frequently initiated at a lower dose of 150 mg/m^2^ and subsequently titrated to full doses after 4 weeks of therapy, depending on patient tolerability. Most responses occur within 2–3 months of treatment initiation but may be delayed. Therefore, in the absence of disease progression or toxicity, treatment should be continued for up to 6 months. For responding patients, treatment should be continued until disease progression and, depending on the quality of the response, adjunctive skin‐directed therapies (e.g., NB‐UVB, PUVA) should be considered [242]. Guidelines describing appropriate laboratory monitoring, supportive care, and safe clinical prescribing of bexarotene have been recently published [241].

#### 4.3.3 HDAC Inhibitors

Histone deacetylases (HDACs) catalyze the removal of acetyl groups from both histone and non‐histone proteins. As histone acetylation is associated with an open chromatin configuration associated with active gene transcription, HDACs contribute to histone deacetylation and the epigenetic repression of gene transcription. As HDACs regulate a wide variety of processes involved in carcinogenesis, multiple mechanisms may explain the clinical activity of HDAC inhibitors [243, 244], including altered gene expression of cell‐cycle and apoptotic regulatory proteins [245, 246, 247, 248, 249], acetylation of non‐histone proteins regulating cell growth and survival [250, 251, 252, 253, 254], angiogenesis [255, 256], aggresome formation [257], and DNA repair [258]. In addition, HDAC inhibitors have profound effects on the tumor microenvironment in CTCL [259].

Vorinostat (suberoylanilide hydroxamic acid, SAHA) and romidepsin (depsipeptide) inhibit class I and II HDACs (i.e., pan‐HDAC inhibitors), the former being widely expressed in various lymphoma subtypes [260]. Early phase I studies of both vorinostat and romidepsin established their safety and potential efficacy in lymphoproliferative disorders, including CTCL [261], thus paving the way for larger phase II studies. An earlier phase II study established 400 mg of oral vorinostat once daily as the optimal dose that was investigated further in 74 previously treated patients with CTCL, most of whom (> 80%) had advanced‐stage disease [262, 263]. The overall response rate was approximately 30% for patients with advanced‐stage disease and was associated with a median duration of response estimated to exceed 185 days. However, it is noteworthy that the reported response rate observed with vorinostat, using updated response criteria, was considerably lower (i.e., < 10%) in MAVORIC [232]. Most responses were rapid (i.e., < 2 months) and were also noted in patients with tumor‐stage disease and Sézary syndrome [264]. Patients who failed to achieve an objective response appeared to derive some clinical benefit, including stable disease, decreased lymphadenopathy, and pruritis relief, with treatment. The most common non‐hematologic adverse events, observed in almost 50% of patients, were gastrointestinal toxicities (nausea, vomiting, diarrhea). Hematologic toxicities, including anemia or thrombocytopenia, were observed in up to 20% of patients. Among responding patients, long‐term therapy with vorinostat appears to be well tolerated [265]. Prolongation of the QT interval was rarely observed, but monitoring and appropriate electrolyte replacement are recommended for those patients at risk for QT prolongation [266].

Romidepsin, administered as a 4‐h intravenous infusion (14 mg/m^2^) days 1, 8, and 15 every 4 weeks, was evaluated in two phase II studies, the largest of which included 96 patients, most with advanced‐stage disease [267, 268]. The overall response rate was 38% for patients with advanced‐stage disease, with a median duration of response that exceeded 1 year. A toxicity profile similar to that described for vorinostat was observed. Intensive cardiac monitoring in a subset of these patients failed to demonstrate any clinically significant cardiotoxicity [269]. A subset of MF/SS patients, after induction with romidepsin at the standard dose, may anticipate a durable remission with attenuated “maintenance” (every 2‐ or 4‐week) dosing. For example, among 38 MF/SS patients, 17 achieved a durable (> 6 month) remission, 9 of whom were maintained with an attenuated, dose‐sparing schedule [270]. Among the patients achieving a durable remission, the median duration of treatment was 15 months (range: 7–34 months).

Additional HDAC inhibitors, including potent pan‐HDAC inhibitors, appear to have activity in CTCL [249, 271, 272]. Further studies are needed to fully define the mechanisms of resistance to HDAC inhibition in CTCL [249, 273, 274, 275, 276, 277], enabling the development of rational therapeutic combinations incorporating HDAC inhibitors in CTCL [278, 279].

#### 4.3.4 Interferons

Interferons (i.e., interferon alpha‐2b, interferon gamma‐1b) have pleiotropic and immunomodulatory effects in CTCL and are associated with overall response rates as high as 50%–70% and a complete response rate of 20%–30%, particularly in patients with limited‐stage disease [280, 281, 282, 283, 284]. While often considered as second‐line therapy for limited‐stage CTCL, interferon‐alpha, frequently at doses ranging from 3 to 10 million units daily to three times weekly, is a treatment to be considered in the first‐line setting in patients with advanced‐stage disease. Responses, which may be achieved within a few months, are observed in patients with tumor‐stage MF and SS and are occasionally durable [205, 285]. Furthermore, interferon‐alpha may be successfully combined with a number of other therapeutic modalities frequently utilized in the management of these patients, including PUVA, bexarotene, chemotherapy, and ECP [286, 287, 288, 289, 290, 291, 292, 293, 294, 295, 296, 297, 298, 299]. For example, in a cohort of 51 mostly advanced‐stage patients treated with single‐agent low‐dose interferon‐alpha, responses were observed in 34 (67%), including 21 (41%) with a complete response and 9 with a long‐term remission [283]. Similarly, in a cohort of 47 patients with stage III/IV disease, 89% of whom had peripheral blood involvement, a response rate exceeding 80% was observed in those treated with a combination of ECP and interferon‐alpha [299]. Interferon‐alpha is associated with myelosuppression, transaminitis, and dose‐limiting flu‐like side effects, particularly at higher doses.

#### 4.3.5 Extracorporeal Photophoresis

During extracorporeal photophoresis (ECP) pooled leukapheresis and plasmapheresis products are exposed to 8‐methoxypsoralen (8‐MOP) prior to extracorporeal circulation through a 1 mm thick disposable cassette exposed to UVA radiation. The irradiated leukocytes, representing approximately 5% of peripheral blood leukocytes, are subsequently reinfused. Psoralen covalently binds and crosslinks DNA following UVA exposure, leading to the induction of apoptosis in the majority of treated lymphocytes by multiple mechanisms involving bcl‐2 family members, disruption of the mitochondrial membrane potential, and extrinsic cell death pathways [300, 301, 302]. In contrast, ECP leads to monocyte activation, including significant changes in gene expression [303], and dendritic cell differentiation, which is thought to culminate in enhanced antigen presentation and the initiation of a host immune response [304].

Following the landmark study by Edelson and colleagues describing responses in 27 out of 37 patients with erythrodermic CTCL treated with ECP, ECP was approved by the Food and Drug Administration of the USA for the treatment of CTCL and is now considered the treatment of choice in the first‐line management of patients with Sézary syndrome in many centers [305]. Furthermore, retrospective series demonstrate that ECP is associated with superior time to next treatment when compared with most systemic therapies, including HDAC inhibitors [206]. While responses vary between case series, overall response rates hover around 60%, with a complete response rate of approximately 20% [306, 307, 308, 309]. As current treatment protocols no longer require the oral administration of 8‐MOP, eliminating nausea, ECP is safe and generally very well tolerated. Long‐term use of ECP may cause iron deficiency anemia due to the small residual blood volume that is not returned to the patient [310]. While the precise mechanism of action is incompletely understood, evidence suggests that ECP has immunomodulatory effects, which may augment host anti‐tumor immunity [311, 312]. It is not surprising then that the median time to response following the initiation of ECP is approximately 6 months. Median survival exceeding 8 years has been observed in ECP treated patients and among complete responders, many experience durable responses which may permit, for some, weaning from CTCL‐directed therapies [306, 313, 314, 315]. In a retrospective study, patients treated with ECP early (i.e., within the first three lines of therapy) experienced superior median time to next treatment (approaching 4 years) when compared to either those treated with alternative agents or ECP later in the course of therapy [316]. While patient‐ or disease‐specific factors which may predict a response to therapy are imperfect [317], Sezary patients without significant nodal or visceral disease who initiate ECP promptly after diagnosis may be more likely to respond. In addition, patients without profound immune deficiencies, reflected by normal or near‐normal cytotoxic T‐cell and CD4/CD8 values and the absence of prior exposure to systemic chemotherapy, may be more likely to respond to therapy [306, 308, 314]. While effective as monotherapy, ECP has also been combined with other therapeutic strategies, including interferon, bexarotene, and TSEBT [289, 299, 313, 318, 319, 320].

#### 4.3.6 Mogamulizumab

Mogamulizumab (KW‐0761) is a humanized monoclonal antibody specific for the chemokine receptor CCR4 that has been defucosylated and is consequently associated with enhanced antibody‐dependent cell‐mediated cytotoxicity (ADCC). In a phase I/2 study, mogamulizumab was well tolerated and was associated with an overall response rate of 37%. A similar response rate of 29% (2/7), all partial, was observed in a phase II Japanese study [321, 322]. In addition to ADCC‐mediated clearance of malignant T cells, mogamulizumab may inhibit T_reg_‐mediated immune suppression [323, 324], and may warrant further investigation with immunomodulatory therapies, including immune checkpoint blockade [325]. A randomized, phase III clinical trial comparing mogamulizumab and vorinostat in relapsed/refractory CTCL (MAVORIC) demonstrated a significant improvement in progression‐free survival among MF/SS patients randomized to mogamulizumab [232]. Overall responses in patients treated with mogamulizumab were higher in the blood compartment (68%) when compared with those observed in the skin (42%) or lymph nodes (17%). Not surprisingly then, the overall response rate was highest among SS patients (37%). Overall, treatment with mogamulizumab was well tolerated, with few ≥ grade 3 adverse events (AE's). Infusion‐related reactions were the most common grade 1 or 2 AE's and were observed in 32%. Mogamulizumab‐associated rashes are observed and may clinically and histopathologically mimic CTCL, but may be managed without discontinuation of therapy [326]. Treatment‐associated rashes are characterized by macrophage‐ and CD8+ T‐cell‐rich infiltrates and have been associated with superior disease control in Sezary patients [327]. These positive findings led to mogamulizumab's approval by the FDA in 2018 for MF/SS patients who have failed at least one prior systemic therapy. In SS, durable remissions (> 12 months) are achievable after treatment discontinuation in patients who have achieved a remission, and treatment can be successfully reinitiated at the time of subsequent relapse [47].

#### 4.3.7 Alemtuzumab

Alemtuzumab is a humanized IgG1 monoclonal antibody directed against CD52, an antigen widely expressed by B‐cells, T‐cells, and monocytes [328]. In a phase II study in 22 patients with advanced‐stage MF/SS, overall and complete response rates of 55% and 32%, respectively, were observed, with a median time to treatment failure of 1 year [329]. Given the significant risk of infectious complications, low‐dose subcutaneous alemtuzumab was investigated in 14 patients with SS, most of whom had relapsed/refractory disease [330]. Most patients in this study received 3 mg of subcutaneous alemtuzumab on day 1, followed by a 10 mg dose on alternating days until the Sézary count was < 1000/mm^3^. With the exception of a single patient whose best response was stable disease, 9 out of 10 patients treated in this manner achieved a response, 3 of which were complete. For most patients, the time to treatment failure exceeded 12 months. What is notable, however, is that infectious complications were not observed in patients treated with the lowest dose (i.e., 10 mg) of alemtuzumab. Similar results, with no infectious complications, were reported in a small cohort of patients treated with modified, low‐dose subcutaneous alemtuzumab for 6 weeks [331]. In addition to hematologic toxicity, conventionally dosed alemtuzumab in advanced‐stage MF/SS is associated with a high incidence of infectious complications [329, 330, 332, 333, 334, 335]. Overall, infectious complications have been observed in two‐thirds of treated patients, most of which are bacterial, including sepsis. Cytomegalovirus (CMV) reactivation is the most common viral infection. In addition, *Pneumocystis jirovecii* pneumonia and invasive fungal infections have also been observed. Therefore, trimethoprim‐sulphamethoxazole and acyclovir should be routinely administered for PJP and HSV/VZV prophylaxis, respectively, in patients receiving alemtuzumab. In addition, CMV surveillance should be performed every 1–2 weeks by quantitative PCR and suppressive therapy with ganciclovir or oral valganciclovir initiated in response to viral reactivation. Low‐dose subcutaneous alemtuzumab appears to be safe and efficacious in selected patients with advanced‐stage MF/SS provided with appropriate supportive care.

#### 4.3.8 Brentuximab Vedotin

Given the promising response rates observed with brentuximab vedotin (BV) in phase II studies [336, 337], a randomized, phase III clinical trial (ALCANZA) comparing BV with an investigator's choice (methotrexate or bexarotene) was performed and demonstrated a significantly improved PFS (> 12 months vs. 3.5 months) for patients randomized to BV, and led to its FDA approval in previously treated CTCL [231, 338]. Among MF patients with limited‐stage disease treated with BV, a response lasting at least 4 months (ORR4) was observed in 40%, whereas an ORR4 of 63% was observed among patients with tumor‐stage (stage IIB) disease. Consistent with prior experience in “CD30 high” lymphomas, an ORR4 of 89% was observed among patients with primary cutaneous ALCL with disease confined to the skin. More recently, and with a median follow‐up of 46 months, the final ALCANZA data confirm the benefit associated with BV. Among MF patients, the median PFS in BV‐treated patients was 16.1 months, compared with 3.5 months in those treated with either methotrexate or bexarotene [230]. Not surprisingly then, 1 year following treatment, 34.5% of BV patients required treatment with an alternative systemic agent, whereas 86.6% of methotrexate or bexarotene treated patients required an alternative therapy. The benefit associated with BV was observed independent of CD30 expression or the presence of large cell transformation [338]. Standard immunohistochemistry may underestimate the extent of CD30 expression [337], and significant intra‐patient variability in CD30 expression over time and across disease sites is anticipated [338, 339]. Furthermore, CD30 expression is dynamic and responses are observed in patients with absent, “low” (< 5%–10%) and “high” CD30 expression [336, 337, 338]. Therefore, therapeutic decision‐making for patients eligible for brentuximab is not contingent on CD30 status.

#### 4.3.9 Denileukin Diftitox‐Cxdl (E7777)

Components of the trimeric IL‐2 receptor complex, comprised of an alpha chain (CD25) required for high‐affinity binding, beta chain (CD122) and a common gamma chain (CD132), are expressed by clonal T cells in CTCL. Targeting the IL‐2 receptor with the monoclonal antibody Daclizumab was safe and partially effective in adult T‐cell leukemia/lymphoma [340, 341]. In hopes of improving upon these results targeting the IL‐2 receptor, denileukin diftitox (Dd) was developed (reviewed in [342]). Denileukin diftitox is a fusion protein comprised of human IL‐2 fused to a truncated diphtheria toxin, which has high affinity for the IL‐2 receptor and is internalized upon receptor binding, leading to liberation of the toxin and the induction of apoptosis. Phase I/II studies demonstrated objective responses in approximately one‐third of patients [343, 344, 345], leading to a phase III study which randomized 71 patients with CD25 positive (i.e., ≥ 20% T cells positive by immunohistochemistry) CTCL, most with advanced‐stage disease, to either 9 or 18 μg/kg/day of Dd given intravenously on five consecutive days every 3 weeks for up to 8 cycles [346]. An objective response, 20% partial and 10% complete, was observed in 30% of patients, while an additional 32% of patients had stable disease. The median time to response was 6 weeks, with 95% of responding patients demonstrating evidence of response by week 9. The median duration of response was 7 months (range 2.7–46.1 months). Among responders, significant improvements in self‐reported quality of life were observed [347]. Results of a large phase III placebo‐controlled trial, utilizing the same Dd dosing schedule, were reported. An overall response rate of 44% was observed and was associated with a median progression‐free survival exceeding 2 years [348]. While only patients felt to have CD25 positive disease were included in these studies, biopsies obtained from different sites or at different times demonstrate significant variability, suggesting that patients with CD25 negative CTCL may benefit from treatment. In a prospective analysis, a significant difference in response rate was noted between CD25 positive and negative lymphomas, with only 20% of patients with absent or low‐level expression responding to treatment, compared with a response rate approaching 80% for those with CD25 positive disease [349]. In a meta‐analysis of three trials, including 307 patients, the overall response rate for Dd‐treated patients that were CD25 positive was 47.5%, and was associated with a median progression‐free survival exceeding 2 years [350]. In contrast, a lower response rate of 30.6%, with a progression‐free survival exceeding 487 days, was observed among CD25 negative patients. For patients given placebo (*n* = 44), the reported response rate was 15.9% and a median progression‐free survival of 4 months was observed. Of note, responses were observed in Dd‐retreated patients who relapsed after achieving an initial response.

A vascular leak syndrome, defined as the development of at least two out of three symptoms, including hypotension, edema, and hypoalbuminemia (albumin < 3 g/dL), has been reported in approximately 25% of patients, usually within the first few cycles of treatment. Most of these patients were retreated without further symptoms. Approximately two‐thirds of patients developed infusion reactions within 1 h of treatment, but they are ameliorated with corticosteroid premedication [351]. More recently, an improved purity denileukin diftitox‐Cxdl (E7777) has completed clinical testing, achieved efficacy results in a phase III study comparable to those historically reported [352], and was licensed by the FDA in 2024 for stage I‐III CTCL failing at least one prior therapy.

#### 4.3.10 Checkpoint Blockade

Durable remissions may be achieved with immunomodulatory therapies, including ECP and interferon‐α. While largely anecdotal, these observations suggest that host immunity, when properly harnessed, can lead to durable responses in selected patients. These observations, coupled with high‐level PD‐L1 expression in a substantial minority of patients and PD‐L1 structural variants in others [353, 354], provide a strong rationale for checkpoint blockade (CPB) [355, 356]. While few CTCL patients have been included in early phase clinical trials, durable responses have been observed (Table 2). For example, in a phase II study in heavily pretreated patients, an overall response rate of 38% was observed in advanced‐stage patients treated with pembrolizumab [359]. MF with large cell transformation (LCT) are genetically complex (with a high mutational burden), frequently downregulate MHC class I [30], and express PD‐L1, including PD‐L1 structural variants [353], all of which are consistent with immune evasion. While the experience in LCT patients is limited, the available evidence supports the utility of checkpoint blockade in these patients [353]. The risk of “hyperprogression” is low in CTCL, as in many solid tumors [361], and the currently available evidence in CTCL is limited to anecdotal reports [362, 363]. In contrast, “tumor flare,” leading to increased skin tenderness and pruritis, which may be difficult to distinguish from disease progression, is common, particularly in patients whose malignant T cells express PD‐1 [359]. Flare reactions should not lead to treatment discontinuation, as they are managed with supportive care, including topical corticosteroids [359]. These encouraging results, in conjunction with the smorgasbord of currently available immunomodulatory agents, lend themselves to future and ongoing combinatorial strategies [356].

**TABLE 2:** Summary of checkpoint (PD‐1/PD‐L1) blockade in CTCL.

Treatment, *n*	Response rate (*n*, %)	Duration of response	References
Atezolizumab, *n* = 26	4 PR (15.4%) 10 SD (38.5%)	Median TTNT 5.9 months	[357]
Durvalumab, *n* = 12	5 (42%)	Median PFS 6 months; 12‐month PFS 36%	[358]
Nivolumab, *n* = 13	2 PR (15%) 9 SD (69%)	24+, 50+ weeks	[354]
Pembrolizumab, *n* = 24	7 PR (29.2%) 2 CR (8.3%) Cutaneous flares noted in 53% of SS, but did not result in treatment discontinuation	Median DOR not reached, median follow‐up 58 weeks	[359]
Pembrolizumab, *n* = 3	1 CR (33%)		[360]
Pembrolizumab, *n* = 3	3 PR/near CR (100%)	> 110 weeks, 9 and 12 weeks	[353]
Total, *n* = 81	Cumulative ORR: 30%		

Abbreviations: CR, complete response; DOR, duration of response; ORR, overall response rate; PR, partial response; SD, stable disease.

#### 4.3.11 Systemic Chemotherapy

Responses to conventional chemotherapeutic agents are rarely durable in CTCL [156], being associated with a median time‐to‐next treatment that is measured in months [205, 206]. Consequently, > 90% of patients treated in this manner will require additional therapy within the first year of therapy. Furthermore, first‐line treatment with systemic chemotherapy has been associated with increased mortality [229]. Therefore, multiagent chemotherapy is rarely utilized. Therefore, novel therapeutic agents, including clinical trial participation, are preferred. As there is no standard of care for patients with MF/SS requiring systemic chemotherapy and the decision to initiate therapy is individualized, including consideration of responses and complications related to prior therapies, participation in a well‐designed clinical trial is always worth consideration.

Pralatrexate, a novel antifolate with a high affinity for the reduced folate carrier (RFC‐1) and a novel mechanism of resistance when compared with methotrexate [364, 365, 366], was associated with an overall response rate of 29% in the PROPEL study. This study was comprised largely of peripheral T‐cell lymphoma patients, most of whom had refractory disease [367]. Notably, 12 patients with transformed MF were included in the study [368]. Many of these patients had received more than five prior systemic therapies, including CHOP or CHOP‐like regimens. With only a single exception, these patients were refractory to their most recent therapy. Responses, as assessed by the study investigators, were observed in 58% of patients with a median duration of response and progression‐free survival of 4–5 months. Results of a dose‐finding study were reported in a larger cohort of CTCL patients [369]. In this study, the optimal dose was identified as 15 mg/m^2^, given weekly for 3 weeks out of 4, and was associated with an overall response rate of 43%. In addition to folic acid and vitamin B12 supplementation [370], oral leucovorin (25 mg, three times daily, on days 2 and 3) significantly reduces the rate of mucositis [371, 372]. Additional therapeutic approaches, including proteasome inhibition [373], immunomodulatory strategies [374], and more targeted approaches warrant further investigation [24, 375].

Single‐agent gemcitabine (1200 mg/m^2^ on days 1, 8, and 15 of a 28 day cycle) is generally well tolerated and associated with an overall response rate of ≈70%, most of which are partial, with a median durability of ≈1 year [376, 377, 378, 379]. Pegylated liposomal doxorubicin, given (20 mg/m^2^) every 2 weeks, has been associated with overall response rates comparable to pralatrexate (≈40%), few of which were complete [380, 381]. More recent real‐life experience suggests a higher response in the skin compartment, including a response rate as high as 76% in patients with tumor‐stage disease, but considerably lower response rates (< 30%) in other compartments [382]. Rare deaths attributed to cardiotoxicity have been reported [380, 381].

#### 4.3.12 Allogeneic Stem Cell Transplantation

The available experience with high‐dose chemotherapy and autologous stem cell transplantation, largely confined to case series, suggests that responses following treatment are transient. In contrast, the durable remissions observed following allogeneic transplantation may be explained by the graft versus lymphoma immune response [383, 384]. A retrospective analysis of 60 patients with advanced‐stage MF/SS who underwent allogeneic stem cell transplantation was recently reported [385]. In this series, patients had received a median of 4 prior therapies prior to undergoing either reduced‐conditioning (73%) or myeloablative (27%) conditioning prior to related (75%) or matched‐unrelated donor (25%) transplantation. Non‐relapse mortality at 1 year was 14% for patients receiving reduced‐intensity conditioning or HLA identical/related donor stem cells and 38%–40% for those undergoing myeloablative conditioning or receiving match‐unrelated donor grafts. Transplantation during an early phase of disease (defined as first or second remission or relapse following 3 or fewer systemic therapies) was associated with lower relapse rates (25% vs. 44% at 1 year) and a statistically insignificant increase in 3‐year overall survival (68% vs. 46%). Given the differences in non‐relapse mortality, both reduced‐intensity conditioning and use of matched‐related donors were associated with superior overall survival (63% at 3 years). Seventeen out of 26 patients who relapsed received donor‐lymphocyte infusions. Of these, 47% achieved a complete remission, thus providing evidence for a graft‐versus‐lymphoma effect in MF/SS. The estimated 3‐year progression‐free and overall survival were 34% and 53%, respectively. A more recent update of the EBMT experience again demonstrates that allogeneic stem‐cell transplantation is curative in a minority of patients, but non‐relapse mortality and disease progression remain challenging [386]. Similar outcomes have been observed in a large series from the CIBMTR (*n* = 129), as non‐relapse mortality and disease progression at 1 year were 19% and 50%, respectively, and 5‐year PFS and OS were 17% and 32%, respectively [387]. A systematic review and meta‐analysis, pooling data from five studies (and 266 patients), demonstrated a relapse rate following allogeneic transplantation of 47% and a non‐relapse mortality rate of 19% [388]. Given the possibility of complete and durable remissions, allogeneic stem‐cell transplantation may be considered in selected patients, and consensus guidelines to aid in patient selection have been recently published [389, 390], but should be performed at experienced academic centers [285, 391, 392].

#### 4.3.13 Clinical Trial Participation

Quality of life (QOL) and supportive care considerations are critically important in treatment selection and CTCL management. Consequently, patients benefit from a multidisciplinary approach, with input provided by an experienced dermatopathologist, dermatologist, and both medical and radiation oncologists. Treatments are frequently provided in an escalated and serial fashion, and in appropriately selected patients, occasionally recycled. While most responses with currently available agents are not complete or durable, stable disease, particularly in patients with limited‐stage disease, may be associated with improved QOL [204]. Improved understanding of disease pathogenesis, including appreciation of both the genetic landscape and the tumor microenvironment's role in CTCL, has identified novel therapeutic vulnerabilities, many of which are the subject of ongoing clinical trials. Consequently, clinical trial participation will continue to play an increasingly important role in the multidisciplinary management of CTCL patients and is strongly encouraged.

## Ethics Statement

The authors have nothing to report.

## Conflicts of Interest

The authors declare no conflicts of interest.

## Data Availability

Data sharing is not applicable to this article as no new data were created or analyzed in this study.
